# 

**Published:** 1915-08-07

**Authors:** 


### CONTENTS.

PAGE

**The Medical Corporations and the West London Hospital** ... ... ... ... ... 383

**A Year of War and Drug Prices** ... ... 384

**Hospital and Institutional News** ... ... ... 385

In St. Paul's Cathedral, August 4, 1915—The Success of Hospital Sunday—The New Zealand Hospital at Walton-on-Thames—Dangers to Attendants in Mental Wards—Mr. Lloyd George and War Service Badges—Six Weeks in a German Internment Camp—Local Congresses and Gifts to Hospitals—London Insurance Committee's Pharmacopœia — Practitioner's Suicide with Veronal—Australia and Her Wounded—Assistant Medical Superintendents' Salaries Increased—Increase of Salary for an Infirmary Superintendent—The Waste of Tobacco Health of the Troops in the Dardanelles—Patent Medicines and Gifts to Hospitals—A Strange Bequest to a Doctor—The Red Cross Motor Launch—An Unfortunate Mistake and Its Lesson—An Army Pharmaceutical Service—A Hero at Westminster Hospital—A Children's Ward for Soldiers—Medical Students and the New Master of Downing—Farm Work for Epileptics—The Seely Memorial at Nottingham —Bank Holiday and the Week's Drug Market —To Our Readers.

**The British Hospitals Association** .. ... 391

A Conversation at Charing Cross Hospital?

The Shortage of Resident Medical Officers.

The Discussion on Mr. Buchanan's Paper.

**A Medical Causerie** ... ... ... ... ... 394

**Round the World** ... ... ... ... ... 395

Fellow-Workers and the Roll of Honour.

**The War and the Hospital Sunday Fund** ... 396

Larger Collections and Increased Grants: The Total of £70,600.

The Distribution and the War—" Hospital-Sunday and You."

Report of the Committee of Distribution to the Council: Awards Recommended by the Committee for the Year 1915.

[*See over.*]

### CONTENTS—(*continued*).

PAGE

**The Editor's Letter-Box** ... ... ... ... 399
The Hospital Egg: Imports and the Contract System.
Economy and the Food Question.

**The School Doctor's Outfit** ... ... ... 400

II.—The Contents of the Doctor's Bag.

**The Institutional Library** ... ... ... ... 401
The History of the Voluntary Aid Detachments: The Men and Women of Kent—The Devon V.A.D.
A Tuberculin Guide.
A List of War Help Societies.
Drugs in the Malay States.

**Personalia** ... ... ... ... ... ... vi

**Leicester Royal Infirmary** ... ... ... ... vi

The War Loan and War Service.

PAGE

**The Royal College of Physicians** ... ... vi

**The Institutional Worker** ... ... (Suppl.) 1
The Disposal of Broken or Disused Material: Answer to July Question.
Practical Matters: Half-Watt Lamps—How to Repair Celluloid—An Ideal Bed-Rest.
Question for August: Surgical Instruments and Appliances.

Enquiries and Answers.
War Matters: Massage and Electrical Treatment for the Wounded—Nursing the Wounded—Treatment for Removal of Vermin.
The Sick and In Need: Mental Hospitals for Paying Patients.
Miscellaneous: Combination Coal- and Gas-Heated Kitchener.
Acknowledgments.

